# Coffee Silverskin and Spent Coffee Suitable as Neuroprotectors against Cell Death by Beauvericin and α-Zearalenol: Evaluating Strategies of Treatment

**DOI:** 10.3390/toxins13020132

**Published:** 2021-02-10

**Authors:** Ana Juan-García, Giovanni Caprioli, Gianni Sagratini, Jordi Mañes, Cristina Juan

**Affiliations:** 1 Laboratory of Food Chemistry and Toxicology, Faculty of Pharmacy, University of Valencia, Av. Vicent Andrés Estellés s/n, Burjassot, 46100 Valencia, Spain; jordi.manes@uv.es (J.M.); cristina.juan@uv.es (C.J.); 2 Laboratory of Food Chemistry, School of Pharmacy, University of Camerino, Via S. Agostino 1, 62032 Camerino, Italy; giovanni.caprioli@unicam.it (G.C.); gianni.sagratini@unicam.it (G.S.)

**Keywords:** beauvericin, α-zearalenol, coffee silverskin, spent coffee, SH-SY5Y cells

## Abstract

Coffee silverskin and spent coffee have been evaluated in a neuroblastoma cell line (SH-SY5Y cells) against beauvericin (BEA) and α-zearalenol (α-ZEL)-induced cytotoxicity with different strategies of treatment. First, the direct treatment of mycotoxins and coffee by-products extracts in SH-SY5Y cells was assayed. IC_50_ values for α-ZEL were 20.8 and 14.0 µM for 48 h and 72 h, respectively and, for BEA only at 72 h, it was 2.5 µM. Afterwards, the pre-treatment with spent coffee obtained by boiling water increased cell viability for α-ZEL at 24 h and 48 h from 10% to 16% and from 25% to 30%, respectively; while with silverskin coffee, a decrease was observed. Opposite effects were observed for BEA where an increase for silverskin coffee was observed at 24 h and 48 h, from 14% to 23% and from 25% to 44%, respectively; however, a decrease below 50% was observed for spent coffee. Finally, the simultaneous treatment strategy for the highest concentration assayed in SH-SY5Y cells provided higher cytoprotection for α-ZEL (from 44% to 56% for 24 h and 48 h, respectively) than BEA (30% for 24 h and 48 h). Considering the high viability of coffee silverskin extracts for SH-SY5Y cells, there is a forthcoming promising use of these unexploited residues in the near future against mycotoxins effects.

## 1. Introduction

Coffee is one of the most worldwide consumed beverages. Once the coffee beans are collected and ready to be used, the co-products that remain constitute an underexploited residue that needs to be discarded. Coffee industry and local producers are developing strategies for this large amount of coffee residues that need to be disposed or get used as a valuable nutritional by-product. There two main generated by-products: silverskin coffee and spent coffee grounds. The first one, silverskin, corresponds to the thin tegument that covers the coffee bean. Once green coffee beans are roasted, this subproduct can be obtained; whereas spent coffee grounds can be produced either at home or in the industry during the process to produce instant coffee [1].

The presence of complex amounts of compounds in coffee is extensive, and it is known that different effects are associated; as in gene expression involved in inflammation, immune system, and metabolic pathways [2,3], myocardial blood flow, and associations with liver fibrosis, depression, etc. [4,5]. An understanding of the physiological effects of coffee is drastically limited by the complexities deriving from two factors: the vast array of components included in the brewed product and the varied effects of each compound. Caffeine is the major and active compound in coffee; however, coffee is also rich in other bioactive substances with a wide array of physiological effects. The list comprises up to 1000 described phytochemicals, comprising phenols, including chlorogenic and caffeic acid, lactones, diterpenes, including cafestol and kahweol, niacin, and the vitamin B3 precursor trigonelline [6]. The central nervous system, vascular endothelium, heart, liver, adipose tissue and muscle are tissues containing adenosine receptors, which are the caffeine-target main action [7,8].

Coffee and coffee products can have contaminant fungi and are almost removed by winnowing. The presence of mycotoxins, mainly ochratoxin A or aflatoxins from *Aspergillus* spp., are concentrated in the shell fraction but can reach coffee. When coffee is harvested, prepared, transported, or stored, it is common to have the growth of filamentous fungi as well as in fermentation and drying, especially where the water activity is very low [9]. Thus, industries are focused in having a good mycotoxin HACCP (hazard analysis critical control points) system to prevent the consumer´s exposure as well as to obtain safer products. On the other hand, the protective effect of other substances in food or diet could have an important role. The higher the amount of polyphenols, the higher the capacity to inhibit the effect of mycotoxins and the higher the antioxidant activity.

Mycotoxin effects are very diverse, and some are associated with membrane lipid disturbances with effects on cholesterol-interacting proteins, lipoprotein metabolism, and membrane apo E/amyloid beta interactions relevant to hypercholesterolemia with close connections to neurological diseases [10]. Lipopolysaccharides/mycotoxin interactions interfere with apolipoprotein E/Aβ peptide interactions and determine neuron survival [11]. Mycotoxins pass through the blood–brain barrier and affect astrocytes and oligodendrocytes, whose significant roles are maintenance of the blood–brain barrier integration and nutritive support for the myelin. Consequently, these toxins render myelin susceptible to degradation by various factors [12]. In this sense, the neuroblastoma SH-SY5Y cell line is an accepted cell model to study most cellular alterations linked to neurodegerative diseases and to ameliorate their effects. To notice that once mycotoxins and natural compounds, both present in food, are orally consumed, only those with specific characteristics related to polarity, affinity, pKa value, etc. will reach the brain.

Different *Fusarium* and *Aspergillus* mycotoxins have been involved in exerting toxicity in primary astrocytes [13,14]; some *Penicillium* mycotoxins produce neurological disorders in animals [15], and others have the possibility of passing through the blood–brain barrier [16]. Regarding beauvericin (BEA) and α-zearalenone (α-ZEL), many studies have evaluated their presence and their cytotoxicity in different in vitro cellular lines but not as a possible factor for the neurodegenerative disease and its cell neuronal effect. Most of the assays related to neurotoxicity are based on experimental models; however, for in vitro testing, the neuroblastoma cell line SH-SY5Y is a good biological model to be used as an alternative. It is worth mentioning that the presence of other food components can suppress or enhance their effect on those cells.

In vitro approaches to give a wider use of coffee by-products and with the perspective of their possible neuroprotection from mycotoxins BEA and α-ZEL are here studied. For this purpose, three strategies of treatment: direct treatment, pre-treatment, and simultaneous treatment were followed in a human neuroblasstoma cell line SH-SY5Y exposed to two coffee by-products extracts (silverskin coffee and coffee spent) and two mycotoxins (BEA and α-ZEL). Results obtained within this study are focused on (i) studying the capacity of coffee by-product extract to protect cells from mycotoxin attack evaluating strategies of treatment and (ii) describing the first step to approach mechanisms of action of mycotoxins at the neuronal level as well as coffee by-products extracts.

## 2. Results

### 2.1. Cytotoxicity of Mycotoxins and Coffee By-Product Extracts in SH-SY5Y Cells

**Figure 1:**
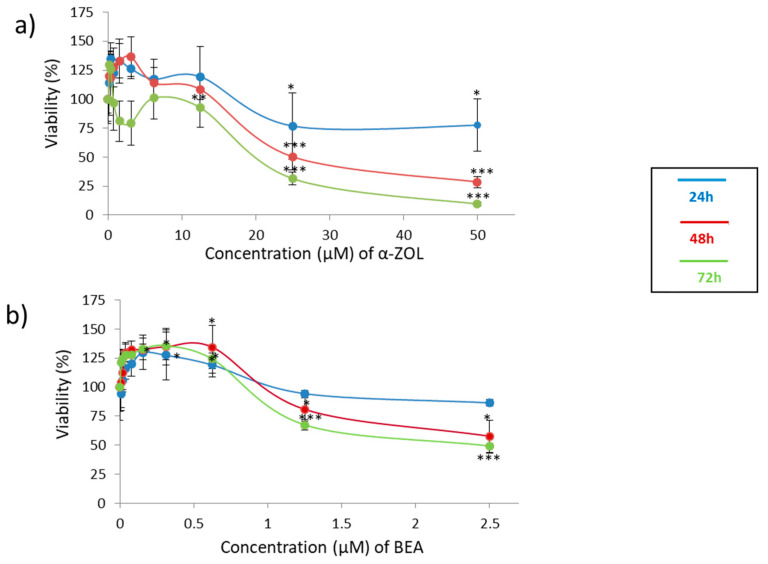
Concentration–effect curves of α-zearalenol (α-ZEL) (**a**) and beauvericin (BEA) (**b**) in SH-SY5Y cells at mycotoxin exposure of 24 h, 48 h and 72 h. The concentration for α-ZEL mycotoxin was 0–50 µM (1:2 dilution), and for BEA, it was 0–2.5 µM (1:2 dilution). * *p* ≤  0.05, ** *p* ≤  0.01 and *** *p*  ≤  0.001 represents significant difference as compared to control values.

The cell viability of α-ZEL and BEA is reported in Figure 1. The results clearly indicated that both mycotoxins have toxic effect on SH-SY5Y cells in a dose and time-dependent manner. Figure 1a shows the cytotoxicity of α-ZEL on SH-SY5Y cells and after 48 h and 72 h of exposure; viability went below 50%, reaching IC_50_ values of 20.8 and 14.0 µM, respectively. It can be observed that α-ZEL exerts a stronger cytotoxic effect in SH-SY5Y cells with respect to BEA. In fact, BEA at 2.5 μM and 72 h cell viability effect is maintained above 50% or very close to it. IC_50_ at 72 h was 2.5 µM for BEA (Figure 1b).

**Figure 2:**
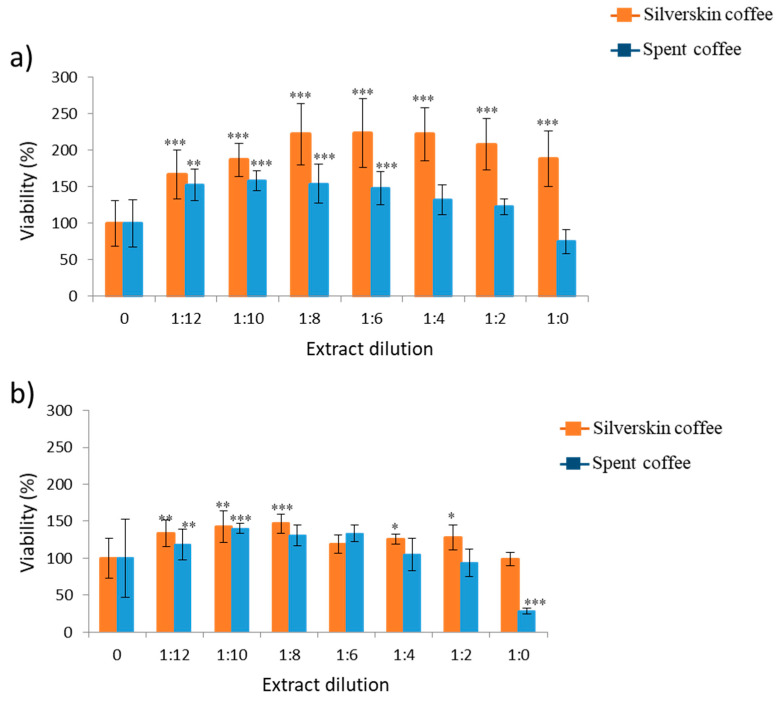
Cytotoxicity of boiling water extract of silverskin (orange bars) coffee and spent coffee (blue bars) on SH-SY5Y cells after 24 h (**a**) and 48 h (**b**) of exposure by (3-(4,5-dimethylthiazol-2-yl)-2,5-diphenyltetrazolium bromide) (MTT) assay. Serial coffee extracts were 1:2 dilutions from 1:0 to 1:12. * p ≤ 0.05, ** p ≤ 0.01 and *** p ≤ 0.001 indicates significant differences compared to the control.

Coffee extracts were evaluated for their cytotoxicity on SH-SY5Y cells at different dilutions (from 1:0 to 1:16) (data published in Juan et al., 2020). Table 1 reports the order for each pure extract (1:0) of coffee by-products assayed in SH-SY5Y cells according to the viability to clearly show that boiling water provided the higher trend of cell viability in SH-SY5Y cells at 24 h and 48 h. Figure 2 collects the viability of serial dilutions for silverskin and spent coffee extract at 24 and 48 h, evidencing that cell viability in coffee silverskin is higher than in spent coffee extract. At 24 h, as reported in Figure 2a, an increase in cell proliferation of 125% for coffee silverskin extract and 62% for spent coffee extract was observed (dilutions from 1:12 to 1:6); however, from 1:4 to 1:0 dilution, extracts showed a significant reduction in cell proliferation of 25% with respect to the control for spent coffee extract and an increase of 100% with respect to the control for coffee silverskin extract (Figure 2a). At 48 h, an increase in cell proliferation of 50% was detected for both coffee extracts by-products (Figure 2b). Dilutions above 1:8 and 1:4 for coffee silverskin extract and spent coffee extract respectively decreased cell viability. This reduction was highly marked for spent coffee until 20% with respect to the control (Figure 2b). As determined by MTT (3-(4,5-dimethylthiazol-2-yl)-2,5-diphenyltetrazolium bromide) assay only after 48 h, spent coffee extract reached IC_50_ values dilution between 1:2 and 1:0. Controls are referred to cells not treated with mycotoxins, which at the same time did not show differences with the cells exposed to solvent control (≤1%).

### 2.2. Cytoprotection Effect of Coffee By-Product Extracts in SH-SY5Y Cells Pre-Treated with α-ZEL and BEA

Boiling water extract was chosen to study cytoprotection, as the direct treatment gave the highest results in cell viability at 1:0 dilution either for 24 and 48 h (Table 1 and Figure 2). Controls refer to cells not treated with mycotoxins, which at the same time did not show differences with the cells exposed to solvent control (≤1%).

#### 2.2.1. Effects of Spent Coffee Boling Water Extract

**Figure 3:**
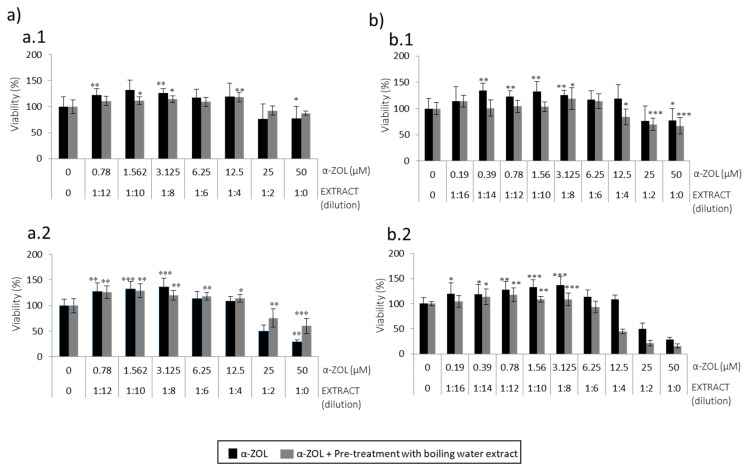
Concentration curves obtained after pre-treatment during 24 h of boiling water extract dilutions of (**a**) spent coffee and (**b**) silverskin coffee, and the subsequent addition of fresh medium with serial dilutions of α-ZEL (starting at 50  µM) during 24 h (**a.1**,**b.1**) and 48 h (**a.2**,**b.2**) in SH-SY5Y cells by MTT assay. All values are expressed as mean ± SD of three replicates (eight wells each time). * *p* ≤ 0.05, ** *p* ≤ 0.01, and *** *p* ≤ 0.001 represent significant difference as compared to control (no treatment).

**Figure 4:**
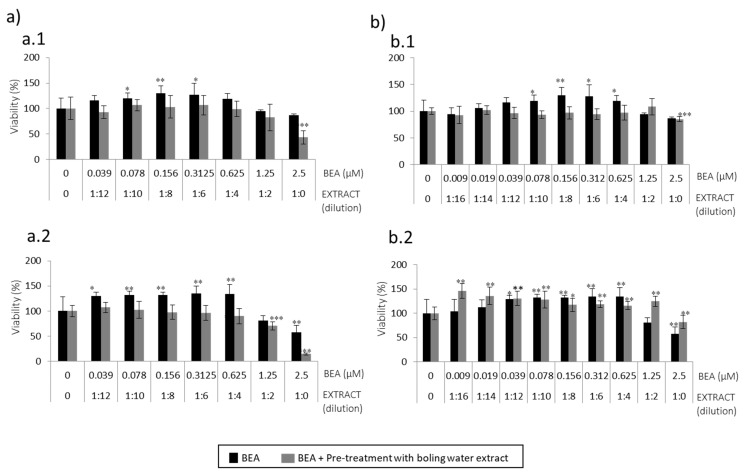
Concentration curves obtained after pre-treatment (24 h) of boiling water extract dilutions (**a**) spent coffee and (**b**) silverskin coffee during 24 h, and subsequent addition of fresh medium with serial dilutions of BEA (starting at 2.5 µM) during 24 h (**a.1**,**b.1**) and 48 h (**a.2**,**b.2**) in SH-SY5Y cells by MTT assay. All values are expressed as mean ± SD of three replicates (eight wells each time). * *p* ≤ 0.05, ** *p* ≤ 0.01, and *** *p* ≤ 0.001 represent significant difference as compared to control (no treatment).

Figure 3a and Figure 4a show how SH-SY5Y cells pre-treated with spent coffee boiling water extract are affected after exposure to α-ZEL and BEA, respectively. SH-SY5Y cell viability decreased after exposure to α-ZEL and BEA (Figure 1). For α-ZEL, cytoprotection was evidenced in two scenarios where the extract was more concentrated (1:2 and 1:0) and mycotoxins had the highest concentration either for 24 h and 48 h (Figure 3(a.1,a.2)). The marked increase of viability ranged from 10 to 16% and 25 to 30% for 24 h and 48 h, respectively respect to the mycotoxin tested alone.

For BEA, such cytoprotection was not achieved, as no pre-treatment with the boiling water extract of spent coffee reached higher viability values as compared to the mycotoxin tested alone either for 24 h or 48 h (Figure 4a). This might be associated to some type of interaction between spent coffee constituents, BEA mycotoxin, and/or the sensibility of SH-SY5Y. Low viability was detected when SH-SY5Y cells were exposed to BEA 2.5 µM pre-treated with pure coffee extract (1:0): 43% and 14% for 24 and 48 h, respectively (Figure 4(a.1,a.2)).

#### 2.2.2. Effects of Coffee Silverskin Boling Water Extract

Figure 3b and Figure 4b show how SH-SY5Y cells pre-treated with coffee silverskin boiling water extract are affected after exposure to α-ZEL and BEA, respectively. For α-ZEL, cytoprotection was not evidenced at 24 h, as viability was maintained very similar in SH-SY5Y cells when pre-treated with coffee extract than with α-ZEL alone (Figure 3(b.1)). Neither did this happen at 48 h, where viability was worst when SH-SY5Y cells were exposed to α-ZEL 50 µM after pre-treatment with pure extract (1:0) viability decreased to 15% (Figure 3(b.2)). It can be hypothesized that there might be some type of interaction with silverskin coffee constituents and α-ZEL mycotoxin.

For BEA, conversely to boiling water of spent coffee extract, cytoprotection was evidenced in the last two scenarios where the extract was more concentrated (1:2 and 1:0) and mycotoxins had the highest concentration either for 24 h and 48 h (Figure 4(b.1,b.2)). The marked increase of viability reached 14% and 44%, for 24 h and 48 h, respectively compared to BEA mycotoxin tested alone.

### 2.3. Cytoprotection Effect of Coffee Silverskin Extracts in SH-SY5Y Cells Treated Simultaneously with α-ZEL and BEA

**Figure 5:**
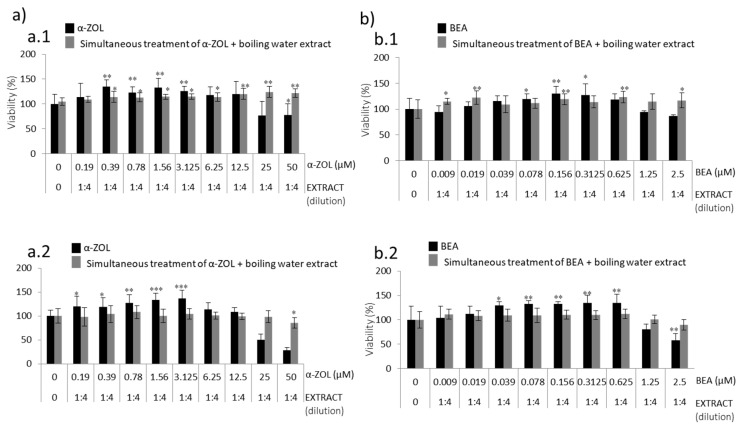
Concentration curves obtained after the simultaneous treatment of boiling water extract (at 1:4 dilution) from silverskin coffee and (**a**) α-ZEL (starting at 50 µM) or (**b**) BEA (starting at 2.5 µM) during 24 h (**a.1**,**b.1**) and 48 h (**a.2**,**b.2**) in SH-SY5Y cells by MTT assay. All values are expressed as mean ± SD of eight replicates. * *p* ≤ 0.05, ** *p* ≤ 0.01, and *** *p* ≤ 0.001 represent significant difference as compared to control (no treatment).

As the highest viabilities in SH-SY5Y cells were reported for coffee silverskin when boiling water extract was tested, the simultaneous treatment assay was performed with this extract with both mycotoxins (α-ZEL or BEA in Figure 5a,b, respectively) in SH-SY5Y cells. The dilution extract was fixed and tested at 1:4, as its viability is maintained close to 100%. Controls refer to cells not treated with mycotoxins, which at the same time did not show any differences with the cells exposed to solvent control (≤1%).

Figure 5a shows the simultaneous treatment of boiling water extract from coffee silverskin and α-ZEL 50 μM 1:2 dilutions at 24 h and 48 h; while Figure 5b shows for BEA 2.5 μM 1:2 dilutions (also for 24 h and 48 h). For α-ZEL and comparing both exposure times, it can be observed that cellular viabilities start to differentiate at 6.25 μM α-ZEL, where there is greater cellular protection (Figure 5a). At both times, when SH-SY5Y cells were exposed to coffee extract 1:4 dilutions and α-ZEL 25 μM and 50 μM simultaneously, the highest protection was reached compared to α-ZEL mycotoxin tested alone as follows: from 48% to 44% and from 40% to 57% for 24 h (Figure 5(a.1)) and 48 h (Figure 5a.2), respectively. For BEA, similar behavior was obtained, and cytoprotection of SH-SY5Y cells was observed for coffee extract 1:4 dilution and BEA at the two highest concentrations tested (Figure 5b). BEA 1.25 μM and 2.5 μM reported a cytoprotection of 20% and 30%, respectively either for 24 h (Figure 5(b.1)) and 48 h (Figure 5b.2) and respect to BEA mycotoxin tested alone. In summary, simultaneous treatment always maintains a higher viability than pre-treatment after 24 h and 48 h for BEA and α-ZEL in SH-SY5Y cells.

**Figure 6:**
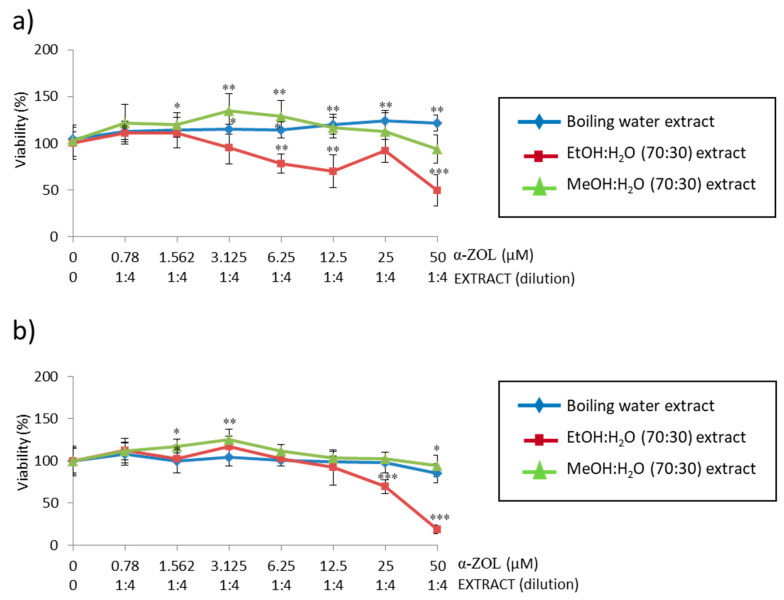
Cytotoxicity of simultaneous treatment of silverskin coffee extracts (boiling water, EtOH:H_2_O (70:30) and MeOH:H_2_O (70:30)) and α-ZEL (starting at 50 µM) on SH-SY5Y cells after 24 h (**a**) and 48 h (**b**) of exposure by MTT assay. Serial coffee extracts was tested at dilution 1:4. * *p* ≤ 0.05, ** *p* ≤ 0.01 and *** *p* ≤ 0.001 indicates significant differences compared to the control.

Lastly, in the simultaneous study, we compared the behavior of boiling water coffee silverskin extract with EtOH:H_2_O (*v*/*v* 70:30) and MeOH:H_2_O (*v*/*v* 40:30) extracts also from coffee silverskin, simulating an identical situation, both at 1:4 fixed extract dilution and α-ZEL (50 μM) mycotoxin and 1:2 dilution (Figure 6).

Viability of 1:4 dilutions of each extract tested alone in SH-SY5Y cells was above or close to 100% (data not shown) for 24 h and 48 h. At 24 h (Figure 6a) after simultaneous treatment with α-ZEL, the viability of the extracts was maintained very similar from the minimum dilution up to α-ZEL 6.25 μM; from this point, the extract EtOH:H_2_O (70:30) is the one that causes a decrease in cellular viability up to a value close to 50% of viability, decreasing at α-ZEL 50 μM until 48%. MeOH:H_2_O (70:30) extract initially generates a higher viability up to α-ZEL 12.5μM; then, it decreases the viability to values below those of the boiling water extract (Figure 6a).

At 48 h (Figure 6b), the situation is very similar to that shown at 24 h, because the cellular viability is almost similar in all three extracts up to α-ZEL 12.5 μM; after this point, the viability of the extract boiling water and MeOH:H_2_O (70:30) is very similar, while there is a noticeable drop with the extract EtOH:H_2_O (70:30), which reaches values of 18%. This means that among all three extracts, at two exposure times, the EtOH:H_2_O (70:30) extract is the one that reflects lesser protection against SH-SY5Y cells simultaneously exposed to α-ZEL mycotoxin.

## 3. Discussion

In the present study, extracts from two coffee by-products (silverskin and spent coffee) were tested by applying different strategies of treatment to evidence whether cytoprotection can be detected in SH-SY5Y mycotoxins treated (α-ZEL and BEA).

There is little literature on mycotoxins in SH-SY5Y cells, and the last report published is for BEA [17]. For zearalenone (ZEA), there is some study on neuronal cells; however, it is the first time that one of its metabolites, α-ZEL, is here studied. The presence of mycotoxins in coffee (grains, brewed, by-products) has been evidenced especially for ochratoxin A, aflatoxins, and fumonisin B2 [18,19,20,21,22]; however, climate change and the importance in recycling and/or reusing residues are redirecting focus to other mycotoxins and the need to study their effect in combination with bioactive compounds.

Despite the lack of studies with mycotoxins in cell lines to report effects of neurotoxicity, the use of alternative models has become crucial. In this sense, the neuroblastoma cell line SH-SY5Y has been used here due to its good characteristics for the exploration of toxicity to humans at this level. Concentrations assayed were chosen according to values of viability reported in MTT cyotoxic assays and close to 80–90% of viability for α-ZEL (see Figure 1a, blue line) and BEA (see Figure 1b, blue line).

The results reported in this study revealed that SH-SY5Y cells respond differently to coffee by-product pure extracts (1:0); nonetheless, the order of higher viability was for most of the scenarios assayed for boiling water (Table 1). In contrast with this, the literature has reported that maximum concentrations of pure coffee silverskin extracts in HepG2 cells were able to reach IC_50_ values [23]. A comparison of two cell lines, Caco-2 and T24 cells, with differences at levels of metabolic capacity, gene expression, differentiation, tumorigenicity, etc. revealed also different sensibility to coffee extracts [24].

Properties associated to coffee grains and brewed coffee have been widely studied, and from the nutritional point of view, the amount of polyphenols as bioactive compounds plays an important role. It has been associated with high amounts of polyphenols with high protecction activity, which results in high cellular proliferation and cytoprotection. This fact was demonstrated for coffee silverskin for most extracts at 1:0 (pure extract) and more specifically for boiling water (Table 1). In a previous study carried out in our lab, total polyphenol content was determined in different extracts. It was revealed that MeOH:H_2_O (70:30) extract from silverskin and spent coffee had the highest amount of polyphenols. When this extract from silverskin was assayed in SH-SY5Y cells, as here reported, its viability fell to the last position at 24 h and 48 h with respect to all the other extracts assayed; in spent coffee extract, it was third and fourth position for 24 h and 48 h, respectively, with respect to the other extracts (Table 1). This is also shown here in Figure 2, where silverskin coffee had viability above spent coffee in a great number of scenarios studied. Related to this, assays of green coffee extracts, which are rich in polyphenols, did not increase proliferation in different cell lines such as A549 cells, MRC5 cells, Caco-2 cells, OE-33, T24, and CCD-8Co [24,25]. The main reason associated is the different activities attributed to polyphenols as reported in other studies.

Three strategies of treatment were assayed in SH-SY5Y cells exposed to coffee by-products extract: direct treatment (Figure 2), pre-treatment followed by mycotoxins´ exposure (BEA and α-ZEL) (Figure 3 and Figure 4), and simultaneous treatment with mycotoxins (BEA and α-ZEL) (Figure 5 and Figure 6). The viability of direct treatment of boiling water coffee silverskin extract in SH-SY5Y cells was above spent coffee: from 14% to 100% and from 14% to 70% for 24 h and 48 h, respectively (Figure 2). Similar to this, researchers observed in HeLA cells with spent coffee decreases in a concentration-dependent manner from 40% to 100% at 2 h and 24 h, respectively (Bravo et al., 2013), although extracts of green and robusta coffee in SH-SY5Y cells did not reveal alterations in cell viability [26].

The second strategy carried out was pre-treatment of SH-SY5Y cells with boiling water coffee by-product extracts to be followed by BEA or α-ZEL (Figure 3 and Figure 4) for 24 h. α-ZEL in SH-SY5Y cells previously treated (pre-treatment) with boiling water spent coffee evidenced a relevant cytoprotection without decreasing viability below 50% at 24 h and 48 h, (Figure 3a). This was not found in pre-treatment with coffee silverskin extract, where viability decreased (10%) with respect to the mycotoxin tested alone at 24 h (Figure 3(b.1)). Viability below 50% was reached only at 48 h from 1:4 (12.5 μM) to 1:0 (50 μM) (Figure 3(b.2)). In summary, pre-treatment of SH-SY5Y cells with boiling water coffee silverskin extract and exposed to α-ZEL acts in a less powerful cytoprotection than with spent coffee extract, as lower viability was reached; this was not verified in the same strategy for spent coffee extract (Figure 3a).

For BEA, it was demonstrated that pre-treatment with boiling water of spent coffee extract was much more toxic than with boiling water coffee silverskin extract (Figure 4). At maximum concentrations assayed and after 24 h and 48 h, values below 50% were reached (Figure 4a); while in silverskin, for some of the points assayed (1:0 extract dilution + BEA 2.5 μM and 1:2 extract dilution + BEA 1.25 μM), the extract protected mycotoxins´ effects, which lead to an increase in viability compared to mycotoxin tested alone (Figure 4b). BEA showed a greater capacity to protect SH-SY5Y cells when coffee silverskin extract is tested than when spent coffee extract is tested (Figure 4). Therefore, BEA cytotoxicity in SH-SY5Y cells is alleviated when pre-treatment with boiling water coffee silverskin extract is tested, but not in the case of spent coffee extract. α-ZEL pre-treated with boiling water spent coffee extract was not toxic, but it was when coffee silverskin extract was tested (Figure 3(b.2)).

The last treatment strategy was based on exposing SH-SY5Y cells simultaneously to boiling water coffee by-products extracts of both BEA or α-ZEL (Figure 5). For these assays, the boiling water coffee by-product extract at 1:4 was selected according to the viability reported in Figure 2 (above 50%) and the saturation of the media; 1:4 dilution was maintained fixed for performing the assays simultaneously. In this simultaneous strategy, the treatment of boiling water coffee silverskin extract reached a high cell viability at 24 h as compared to the mycotoxin tested alone in two points (2.5–1.25 μM for BEA and 50–25 μM for α-ZEL) (Figure 5(a.1,b.1)); but after 48 h, only α-ZEL decreased the viability below 50% (Figure 5(a.2)). Comparing the simultaneous treatment of boiling water coffee silverskin extract for both mycotoxins at the highest concentration assayed, it can be observed that simultaneous treatment with α-ZEL (50 μM) exerts greater cellular protection from 44% to 56% with respect to mycotoxin tested alone for 24 h and 48 h (Figure 5a), respectively; while for BEA, the protection was 30% greater with respect to mycotoxin tested alone for both exposure times (Figure 5b).

In a step further in studying the simultaneous strategy of α-ZEL in SH-SY5Y cells, viability was measured and compared with two more coffee silverskin extracts: MeOH:H_2_O (70:30) and EtOH: H_2_O (70:30) (Figure 6). When comparing these scenarios, boiling water extract kept having the highest viability after 24 h; but after 48 h, it decreased and became higher for MeOH:H_2_O (70:30) extract; EtOH:H_2_O (70:30) showed the lowest viability both at 24 h and 48 h. Ethanolic extract caused a decrease in cellular viability up to a value slightly lower than the IC_50_ at both exposure times. This means that among all three extracts compared, EtOH:H_2_O (70:30) extract does not exert cytoprotection in SH-SY5Y cells following the effect of α-ZEL.

Although there are no previous studies in this regard, investigating mycotoxins exposure to polyphenols and solvent extracts, both pre-treatment and simultaneous treatment had been carried out for evaluating mycotoxins effects. Both have been followed for studying goji berry extracts rich in carotenoids in Caco-2 cells against BEA cytotoxicity [27,28] as well as in HepG2 cells for lentils extracts rich in soyasaponins against alternariol (AOH) cytotoxicity [29]. It reveals that it is a good methodology for evaluating cytoprotection and the potential effects in vitro of bioactive natural compounds.

The effects of antioxidant polyphenolic compounds in cell lines can be found in the literature, but this work is the first to evaluate coffee by-products extracts, silverskin and spent coffee, in SH-SY5Y cells with different strategies of treatments implemented simultaneously and following mycotoxins´ treatment. The results revealed that SH-SY5Y cells respond differently to strategies of treatment and coffee by-product pure extracts (1:0). Nonetheless, it is also evidenced that boiling water extract does not have the entire ability to inhibit the effect of BEA and α-ZEL in all strategies for all scenarios tested, although it was successful for some of them. These facts open the possibility of how to evaluate natural compounds and their potential use from the neuroprotective point of view, and it is starting to captivate the attention of the scientific community. Future perspectives of these studies ought to dive into the capacity of coffee by-products to prevent the formation of reactive oxygen species, while the re-use of these residues contributes to the efficiency of the food industry, being eco-friendly and framed positively within the 2030 Agenda.

## 4. Materials and Methods

### 4.1. Chemicals and Reagents

The reagent grade chemicals and cell culture components used were Dulbecco’s Modified Eagle’s Medium-F12 (DMEM/F-12), fetal bovine serum (FBS), and phosphate buffer saline (PBS), which were supplied by Thermo Fisher, Gibco ™ (Paisley, UK). Methanol (MeOH, HPLC LS/MS grade) was obtained from VWR International (Fontenay-sous-Bois, France). Dimethyl sulfoxide was obtained from Fisher Scientific Co, Fisher BioReagents™ (Geel, Belgium). 3-(4,5-dimethylthiazol- 2-yl)-2,5-diphenyltetrazolium bromide (MTT) for MTT assay, penicillin, streptomycin, and Trypsin–EDTA was purchased from Sigma-Aldrich (St. Louis, MO, USA). Deionized water (<9, MΩcm resistivity) was obtained in the laboratory using a Milli-QSP^®^ Reagent Water System (Millipore, Beadford, MA, USA). The standard of BEA (MW: 783.95 g/mol) and α-ZEL (MW: 320,38 g/mol) were purchased from Sigma-Aldrich (St. Louis, MO, USA). Stock solutions of mycotoxins were prepared in methanol (MeOH) (α-ZEL) and dimethyl sulfoxide (DMSO) (BEA) and maintained at −20 °C in the dark. The final concentration of mycotoxins´ solvents in the medium was ≤1% (*v*/*v*) as per established. All other standards were of standard laboratory grade.

### 4.2. Cell Culture

Human neuroblastoma cell line, SH-SY5Y, was obtained from American Type Culture Collection (ATCC, Manassas, VA, USA) and cultured in Dulbecco’s Modified Eagle’s high-glucose medium (DMEM-HAMF-12 4 g/mL), supplemented with 10% fetal bovine serum (FBS) 100 U/mL penicillin, and 100 mg/mL streptomycin. The cells were sub-cultivated after trypsinization once or twice a week and suspended in complete medium in a 1:3 split ratio. Maximum cell passage was 20. Cells were maintained as monolayer in 150 cm^2^ cell culture flasks with filter screw caps (TPP, Trasadingen, Switzerland). Cell cultures were incubated at 37 °C, 5% CO_2_ atmosphere.

### 4.3. Coffee By-Product Extracts Rich in Polyphenols

Coffee by-products (coffee silverskin and spent coffee) were received from Camerino (Marche-Umbria Reggio, Italy). Five different solvents and mixtures were used for obtaining extracts from 1 and 10 g of coffee silverskin and spent coffee, respectively. Extracts were prepared by using boiling water, alcohol (MeOH), and hydro-alcoholic mixtures: MeOH:H_2_O (*v*/*v*, 70:30), MeOH:H_2_O (*v*/*v*, 50:50), and EtOH:H_2_O (*v*/*v,* 70:30). Final volumes of 50 mL were collected and concentrated until dryness in a Rotary Evaporator Model R-200 (Büchi, Cornaredo, Italy). The dried coffee extract was dissolved in MeOH and placed into topacium vials, which were previously filtered through a nylon filter 0.22 μm pore size (Analysis Vinicos S.L. Tomelloso, Spain). Extracts were preserved at 4 °C until use.

Extract of coffee by-products were analyzed to determine and identify polyphenols (details of amounts in [30]). These assays were carried out previously in our lab by using Folin–Ciocalteau’s reagent [31] and an ultra-high-performance Accurate-Mass Q–TOF-LC/MS analysis (Agilent Technologies, Santa Clara, CA, USA) (see Supplementary Materials Table S1 for details).

### 4.4. MTT Assay

The MTT assay determines the viability of cells by the reduction of yellow soluble tetrazolium salt (MTT), only in the metabolically active cells, via a mitochondrial-dependent reaction to an insoluble purple formazan crystal. Briefly, after exposure to α-ZEL, BEA, and coffee by-product extracts (from silverskin coffee and spent coffee) by direct treatment, pre-treatment, or simultaneous treatment strategies (described in detail in sections below), the medium containing these compounds was removed and cells of each well received 200 µl fresh medium plus 50 µL of MTT. The plates were wrapped in foil and incubated for 4 h at 37 °C. Afterwards, the medium containing the MTT was removed, and the resulting formazan salt was solubilized in DMSO. The absorbance was measured at 570 nm using an ELISA plate reader Multiscan EX (Thermo Scientific, MA, USA).

### 4.5. Strategies of Treatment with Beauvericine, α-Zearalenol, and Coffee By-Product Extracts in SH-SY5Y Cells

SH-SY5Y cells were seeded in 96-well culture plates at 2 × 10^4^ cells/well and set for 24 h before performing the assays with coffee by-products extracts and mycotoxin´s additions. Subsequently, three different strategies were carried out: direct treatment, pre-treatment, and simultaneous treatment, as explained below.

#### 4.5.1. Strategy of Direct Treatment

Cells seeded in 96-well/plates were treated individually with α-ZEL (from 50 to 0.2 µM) or BEA (from 2.5 to 0.005 µM) at 1:2 serial dilution or coffee extracts (five coffee silverskin extract and five spent coffee extract) starting at the dilution ratio of 1:0 and serial dilutions (until 1:16). When 1:0 dilution was used, the amount of the extract was added to the cell media respecting the proportion of 1% of the entire volume of the well. MTT assay was performed after 24 h and 48 h of exposure. Culture medium without extracts and with 1% solvent were used as control.

#### 4.5.2. Strategy of Pre-Treatment

Pre-treatment consisted of a first step of direct exposure to coffee by-product extracts, as described in previous sub-section. After 24 h, extracts were removed, and the dilution of mycotoxins were added at the same concentration as described previously starting at 50 μM for α-ZEL and at 2.5 μM for BEA 1:2 dilutions all along the entire 96-well plate. MTT assay was performed after 24 h and 48 h. Coffee extracts assayed were both coffee silverskin and spent coffee obtained by a boiling water extraction procedure and subsequent treatment with α-ZEL or BEA. Boiling water extract was selected to follow this strategy, as it gave better viability (protection) and opened the possibility of being used further in the food industry.

#### 4.5.3. Strategy of Simultaneous Treatment

Considering the results obtained in our lab and in Section 2.2.1, dilution used for simultaneous treatment was set at 1:4 for coffee extract by-products, which mantained cell viability above 95%. Dilutions above 1:4 started to decrease cell viability. Then, SH-SY5Y cells were simultaneously treated with boiling water coffee extract (silverskin coffee and spent coffee) at the dilution concentration ratio of 1:4 and mycotoxins at a maximum concentration of 50 μM for α-ZEL and 2.5 μM for BEA and diluted 1:2 for the entire 96-well plate. Controls used were the same as those reported in the “*Direct treatment*” Section. After 24 h and 48 h, the MTT assay was performed. Two more silverskin coffee extracts in simultaneous treatment were compared with boiling water: MeOH:H_2_O (70:30, *v*/*v*) and EtOH:H_2_O (70:30, *v*/*v*), with α-ZEL mycotoxin. As mentioned before, extracts tested in such treatment were those that gave better viability (protection) or that could report a possibility of using them further in the food industry.

### 4.6. Statistical Analyses of Data

Statistical analysis of data was carried out using IBM SPSS Statistic version 24.0 (SPSS, Chicago, IL, USA) statistical software package. Data were expressed as mean ± SEM of four independent experiments. The statistical analysis of the results was performed by Student’s t-test for paired samples. Differences with respect to the control group were statistically analyzed using ANOVA followed by the Tukey HSD post hoc test for multiple comparisons; *p* ≤ 0.05 was considered statistically significant.

## Figures and Tables

**Table 1:** Viability ranking (order) of coffee by-product pure extracts (at 1:0) exposed to SH-SY5Y cells (boiling water, MeOH, MeOH:H_2_O (*v*/*v*, 50:50), EtOH:H_2_O (*v*/*v*, 70:30), and MeOH:H_2_O (*v*/*v*, 70:30) at 24 h and 48 h. Gray degradation colors have been assigned for each extract assayed.

	Silverskin Coffee Extracts		Spent Coffee Extract	
Viability Ranking	24 h	48 h	24 h	48 h
1st	Boiling water	Boiling water	Boiling water	EtOH:H_2_O (70:30)
2nd	MeOH:H_2_O (50:50)	EtOH:H_2_O (70:30)	EtOH:H_2_O (70:30)	MeOH
3rd	MeOH	MeOH	MeOH:H2O (70:30)	Boiling water
4th	EtOH:H_2_O (70:30)	MeOH:H_2_O (50:50)	MeOH:H_2_O (50:50)	MeOH:H_2_O (70:30)
5th	MeOH:H_2_O (70:30)	MeOH:H2O (70:30)	MeOH	MeOH:H_2_O (50:50)

## Data Availability

Not applicable.

## Supplementary Materials

The following are available online at https://www.mdpi.com/2072-6651/13/2/132/s1, Table S1: analysis of polyphenols by QTOF –LC/MS.
