# Supplementary material for: Coffee Silverskin and Spent Coffee Suitable as Neuroprotectors against Cell Death by Beauvericin and α-Zearalenol: Evaluating Strategies of Treatment
Source: Toxins (Basel). 2021 Feb 10;13(2):132. doi: 10.3390/toxins13020132 (PMC7916764; doi:10.3390/toxins13020132)
Supplement: Supplementary file 1 [file toxins-13-00132-s001.pdf]

Supplementary Materials: Coffee Silverskin and Spent Coffee Suitable as Neuroprotectors against Cell Death by Beauvericin and α-Zearalenol: Evaluating Strategies of Treatment.

Ana Juan-García, Giovanni Caprioli, Gianni Sagratini, Jordi Mañes and Cristina Juan

Polyphenols were identified using a 1200 Infinity series LC system from Agilent Technologies (Santa Clara, CA, USA) with binary pump, an automatic injector, and coupled to 6540 UHD Accurate-Mass Q-TOF-LC/MS, device equipped with an electrospray ionization Agilent Technologies Dual Jet Stream ion source (Dual AJS ESI) operating in negative ionization mode.

The chromatographic separation of the analytes was conducted at 25 °C with a reversed phase analytical column Agilent InfinityLab Poroshell 120 EC-C18 (3 × 100 mm, 2.7 µm) fitted with an InfinityLab Poroshell 120 EC-C18 (3 × 5 mm, 2.7 µm) guard column. The injection volume was 10 µL. The mobile phase consisted of 0.1% formic acid in water milli-Q (solvent A) and acetonitrile (solvent B) at a flow rate of 0.5 mL/min. The mobile phase gradient (5–95% B) steps were applied as follows: 0–10 min, 5% B; 10–13 min, 95% B; 13–15 min, 95% B. Total run time was 15 min.

Q-TOF-MS conditions used were as follow: drying gas temperature 370 °C with a flow (N_2) of 12.0 L min^{-1}; the pressure on nebulizer was 45 psi; capillary voltage was 3500 V; fragmentor voltage, 110 V; skimmer voltage 65 V and octopole RF peak, 750 V. Agilent Dual Jet Stream electrospray ionization (Dual AJS ESI) interface was used in negative ionization mode and negative ions were acquired in the range of *m*/*z* 100–1100 for MS scans, and *m*/*z* 50–600 for auto MS/MS scans, at a scan rate of 5 scans/s for MS and 3 scans/s for MS/MS, respectively for three different collision energy (20, 30 and 40 eV). Internal mass correction was enabled, by using two reference masses at *m*/*z*. 121.0509 and 922.0098 Instrument control and data acquisition were performed using Agilent MassHunter Workstation software B.08.00. All the MS and MS/MS data of the validation standards were integrated by MassHunter Quantitative Analysis B.10.0 (Agilent Technologies). Table 1 reports the mass measurement parameters of the protonated molecule of polyphenol in coffee by-product extracts. The errors obtained for all compounds were <−2 ppm.

Table S1. LC-qTOF-MS accurate mass measurements in boiling water coffee by-products extract.

Polypohenol	Rt (min)	Molecular Weight	Mass Theoretical (*m*/*z* M-H⁻)	Mass Observed (*m*/*z* M-H⁻)	MS Fragments (*m*/*z* M-H⁻)	Mass Error (ppm)	Score
Chlorogenic acid	4.69	354.0957	353.0872	353.0882	191.0543/127.0397	−2.74	99.69
Gallic acid	4.34	170.0216	169.0147	169.0143	127.1116/71.0511	2.36	99.35
Salicylic acid	5.13	138.0321	137.0245	137.0249	93.0351/65.0405	−2.91	99.41
Vanillic acid	6.39	168.0425	167.0359	167.0352	108.0218/123.0458	4.19	97.11
Vanillin	4.47	152.0473	151.0382	151.0381	146.7437/136.0160	0.66	96.44
1-2-dihydroxybenzene	3.43	110.0367	109.0323	109.0320	100.1120/68.9961	2.75	99.69
3-4-dihydroxyhydrocin-namic	4.26	182.0586	181.0518	181.0514	108.00/134.0369	2.20	86.68
Hydroxibenzoic acid	2.95	138.2037	137.0310	137.0311	100.9328/55.0189	−0.72	93.44
Caffeic acid	5.67	180.0431	179.0353	179.0359	135.0447/107.0506	−3.35	95.32
Dl-3-phenyllactic acid	6.89	166.0636	165.0562	165.0564	145.3990/128.8778	−1.21	99.71
Ferulic acid	6.51	194.0587	193.0513	193.0514	149.4377/96.9620	−0.51	96.93
P-coumaric acid	6.58	164.0475	163.0408	163.0405	159.1024/111.9937	1.84	97.23
Protocatechuic	2.89	154.0267	153.0192	153.0195	112.9856/71.0138	−1.96	99.39
Sinapic acid	4.11	224.0683	223.0615	223.0612	191.0210/149.0232	1.34	99.34
Syringic acid	4.39	198.0531	197.0455	197.0457	123.0083/147.8891	−1.01	98.26
